# PR1-Specific T Cells Are Associated with Unmaintained Cytogenetic Remission of Chronic Myelogenous Leukemia After Interferon Withdrawal

**DOI:** 10.1371/journal.pone.0011770

**Published:** 2010-07-26

**Authors:** Shreya Kanodia, Eric Wieder, Sijie Lu, Moshe Talpaz, Gheath Alatrash, Karen Clise-Dwyer, Jeffrey J. Molldrem

**Affiliations:** 1 Section of Transplant Immunology, Department of Blood and Marrow Transplantation, University of Texas M. D. Anderson Cancer Center, Houston, Texas, United States of America; 2 Department of Experimental Therapeutics, University of Texas M. D. Anderson Cancer Center, Houston, Texas, United States of America; New York University, United States of America

## Abstract

**Background:**

Interferon-α (IFN) induces complete cytogenetic remission (CCR) in 20–25% CML patients and in a small minority of patients; CCR persists after IFN is stopped. IFN induces CCR in part by increasing cytotoxic T lymphocytes (CTL) specific for PR1, the HLA-A2-restricted 9-mer peptide from proteinase 3 and neutrophil elastase, but it is unknown how CCR persists after IFN is stopped.

**Principal Findings:**

We reasoned that PR1-CTL persist and mediate CML-specific immunity in patients that maintain CCR after IFN withdrawal. We found that PR1-CTL were increased in peripheral blood of 7/7 HLA-A2+ patients during unmaintained CCR from 3 to 88 months after IFN withdrawal, as compared to no detectable PR1-CTL in 2/2 IFN-treated CML patients not in CCR. Unprimed PR1-CTL secreted IFNγ and were predominantly CD45RA±CD28+CCR7+CD57-, consistent with functional naïve and central memory (CM) T cells. Similarly, following stimulation, proliferation occurred predominantly in CM PR1-CTL, consistent with long-term immunity sustained by self-renewing CM T cells. PR1-CTL were functionally anergic in one patient 6 months prior to cytogenetic relapse at 26 months after IFN withdrawal, and in three relapsed patients PR1-CTL were undetectable but re-emerged 3–6 months after starting imatinib.

**Conclusion:**

These data support the hypothesis that IFN elicits CML-specific CM CTL that may contribute to continuous CCR after IFN withdrawal and suggest a role for T cell immune therapy with or without tyrosine kinase inhibitors as a strategy to prolong CR in CML.

## Introduction

Since the introduction of interferon-α (IFN) as a treatment for CML [1], [2], randomized trials have shown that it is able to induce hematological remission in 70–80% cases and cytogenetic remission in 35–55% cases [3]. IFN also induces complete cytogenetic remission (CCR) in 13% of patients, of which over approximately 50% are durable for 2–8 years [4]. Studies have shown that IFN-treated chronic phase patients in complete or sustained cytogenetic remission (CR) still have significant (1–12%) numbers of BCR-ABL positive cells [5], [6], [7], identified by fluorescence *in situ* hybridization (FISH) or by quantitative real time-PCR (QR-PCR) of the BCR-ABL fusion gene [6], [8], [9]. In patients with blast crisis CML, CFU-BM may be the reservoir of leukemia stem cells (LSCs) [10]. These cells are not likely to be eliminated by traditional chemotherapy or by tyrosine kinase inhibitors [11]. Conversely, after IFN is discontinued, a small number of patients remain in cytogenetic or molecular remission for months to years without treatment [12], suggesting possible elimination of CML, or the persistence of residual undetectable LSC, which may be intrinsically constrained, controlled by indirect mechanisms that suppress growth, or both. Previous studies showed that IFN directly inhibits CML cells [13], [14], although this mechanism cannot explain persistence of cytogenetic or molecular remission many years after stopping IFN. As an alternative mechanism, IFN induces specific immunity against CML and T cells specific for leukemia-associated antigens (LAA) can target leukemia progenitors and contribute to CR[15], [16]. These observations suggest that long-term leukemia-specific immunity may prevent future outgrowth of CML or may even eliminate leukemia. It is not possible to predict which patients will continue in CR after stopping IFN, and previous studies have not examined such patients to determine whether immunity to LAA persists after IFN withdrawal.

One such LAA is the HLA-A2-restricted PR1 peptide (VLQELNVTV), which is derived from proteinase 3 (P3) and neutrophil elastase (NE), differentiation stage-specific serine proteinases stored in azurophil granules of polymorphonuclear leukocytes [17]. P3 is over-expressed in a variety of myeloid leukemias, including 75% of CML patients, [18] and may be involved in the process of leukemia transformation or maintenance of the leukemia phenotype [19] via the proteolytic regulation of the cyclin dependent kinase inhibitor p21^waf1^ [20]. P3 is expressed in CML progenitors and PR1-specific cytotoxic T lymphocytes (CTL) kill leukemia cells [21] and inhibit HLA-A2^+^ CML colony forming units in proportion to over-expression of P3 in leukemia cells as compared to normal bone marrow cells [15]. Interestingly, the expression of P3 and NE in CD34+ CML cells correlated with improved clinical outcomes after treatment with allogeneic stem cell transplantation or IFN therapy, potentially due to improved PR1-specific anti-leukemia effects [22], [23]. Importantly, PR1-CTL are increased and contribute to CCR in CML patients receiving IFN, but they are not detected in patients at relapse despite continuous treatment with IFN [16]. In addition, PR1-CTL expressing either high or low avidity T cell receptors can be expanded from healthy donor peripheral blood and TCR avidity correlates with CTL effector function [24], similar to T cell immunity to foreign antigens [25], [26], [27], [28]. Importantly, CML cells that overexpress P3 can shape host immunity by inducing apoptotic deletion of high avidity PR1-CTL, which results in loss of functional immunity to CML [24]. Moreover, both P3 target antigen and PR1-CTL were found to be increased in CML patients that continued in CCR while receiving IFN as a single agent maintenance therapy after first achieving CCR during upfront treatment with IFN plus the bcr-abl tyrosine kinase inhibitor imatinib mesylate [29]. This suggests that continuous IFN treatment may maintain CCR by increasing PR1-CTL immunity, although the effect of further IFN withdrawal on persistent PR1-CTL immunity and CCR status is not known.

Therefore, we hypothesized that functional memory PR1-CTL may persist in some patients via long-term self-renewal mechanisms that work to maintain CCR after IFN withdrawal. Here we report that PR1-specific CTL are increased and persist in the peripheral blood of CML patients during unmaintained CCR after stopping IFN and that PR1-CTL secrete IFNγ. Although surprisingly enriched in phenotypically naïve cells, the central memory (CM) PR1-CTL subset showed robust antigen-induced proliferation. Our data suggest that IFN may induce the expansion of memory PR1-CTL with self-renewing capacity in a subset of patients in unmaintained CCR, which may be critical for effective long-term immunity to CML, thereby sustaining CCR in CML patients after IFN withdrawal.

## Materials and Methods

### Ethics Statement

All donors and patients were enrolled on a study protocol that was reviewed and approved by the University of Texas M. D. Anderson Cancer Center (MDACC) Institutional Review Board and provided written informed consent to participate.

### Patients

Seven patients that had an established diagnosis of chronic phase CML based on presence of Philadelphia chromosome, were HLA-A2^+^ and in complete cytogenetic remission (CCR) that was induced by IFN treatment, and two chronic phase CML patients that did not respond to IFN were studied (**Table 1**). Three patients (patients 3, 4, and 6) had a molecular CR documented by absence of *BCR-ABL* transcripts by quantitative real time-polymerase-chain-reaction (QR-PCR), standardized in the Department of Hematopathology at M. D. Anderson Cancer Center. RNA was analyzed by QR-PCR to quantitate *BCR-ABL* and *ABL* transcripts and transcript levels reported on the International Scale (IS). Cytogenetic relapse occurred in 3 patients (patients 2, 6, and 7) after IFN withdrawal, which was treated with imatinib monotherapy, 400 mg daily. Cytogenetic response was assessed by G-banding and counting Ph-positive metaphases in at least 20 cells in metaphase per bone marrow biopsy sample. Median disease duration was 5 years (range of 1 to 7 years). Patients who had previously discontinued IFN treatment, most often due to constitutional side effects, and who remained in CCR, were selected for this study. Prior to study, patients had not received any treatment for CML for 6–88 months.

**Table 1:** Baseline patient characteristics.

Patient Number	Sex	Age	Sokal^1^ score	White cells (×10^−3^/mm^3^)	Platelets (×10^−3^/mm^3^)	ECOG^2^ performance status
1	F	63	Low	14	118	1
2	M	46	Int	89	365	2
3	M	52	Low	23	480	1
4	F	28	Low	46	192	1
5	F	32	Low	18	258	0
6	M	53	Int	69	598	1
7	F	55	Int	112	742	2
8	M	62	Low	38	633	1
9	F	59	High	183	879	3

1 The Sokal score is based on age, spleen size, peripheral blood platelet count, and blast count. Patients are classified as being low-risk (Sokal score <0.8), intermediate-risk (0.8 to 1.2), or high-risk (>1.2).

2 The Eastern Cooperative Oncology Group (ECOG) performance status is graded on a scale of 0 to 5, with 0 indicating that the patient is fully active and able to carry on all pre-disease activities without restriction and 5 indicating that the patient has died.

Characteristics of patients at the time of newly diagnosed Philadelphia chromosome-positive (Ph+) chronic-phase CML.

After informed consent, peripheral blood mononuclear cells (PBMC) from patients were obtained at various time points during their routine clinic follow up, approximately every six months. The cells were separated using Ficoll-Histopaque gradient-density (Organon Teknika Corp., Durham, North Carolina, USA) and subsequently cryopreserved in RPMI-1640 complete medium (CM) (25 mM HEPES buffer, 2 mM L-glutamine, 100 U/ml penicillin, 100 µg/ml streptomycin; Life Technologies Inc., Gaithersburg, Maryland, USA) supplemented with 20% heat-inactivated pooled human AB serum (AB; Sigma-Aldrich, St. Louis, Missouri, USA) and 10% DMSO, according to standard protocols. Before use, cells were thawed at 37°C, washed and suspended in CM plus 10% AB.

### HLA Testing

HLA-A2 expression was confirmed with the BB7.2 antibody, which is specific for the HLA-A*0201 allele. One million PBMC were washed 3 times in PBS and stained with the BB7.2 antibody for 25 minutes on ice, washed again and stained with FITC-labeled goat-anti-mouse secondary antibody (Becton Dickinson Immunocytometry Systems, San Jose, California, USA) for 30 minutes at 4°C in the dark. Cells were washed twice in PBS and re-suspended in PBS +1% paraformaldehyde (PFA). All samples were acquired within 24 hours of staining, using a FACScan flow cytometer and CellQuest software. At least 25000 events were collected.

### Peptide Synthesis

PR1 (aa 169–177) peptide (VLQELNVTV) is derived from the azurophilic granule protein proteinase 3. The cytomegalovirus (CMV) peptide (NLVPMVATV) is derived from pp65, the immunodominant antigen from CMV. Both peptides were synthesized by the MD Anderson Cancer Center Synthetic Antigen Laboratory (Houston, Texas) to a minimum of 95% purity as measured by high performance liquid chromatography.

### Tetramer Synthesis

A 15-amino acid substrate peptide (BSP) for BirA-dependent biotinylation has been engineered onto the C-terminus of HLA-A2. The A2-BSP fusion protein and human β_2_-microglobulin (β_2_M) were expressed in Escherichia coli, and were folded in vitro with the specific peptide ligand. The properly folded MHC-peptide complexes were extensively purified using FPLC and anion exchange, and biotinylated on a single lysine within the BSP using the BirA enzyme (Avidity, Denver, Colorado). Tetramers were produced by mixing the biotinylated MHC-peptide complexes with phycoerythrin (PE)-conjugated Neutravidin (Molecular Probes, Eugene, Oregon) at a molar ratio of 4∶1. PR1 tetramers were validated by staining PBMC from patients immunized with the PR1 peptide. CMV tetramers were validated by staining PBMC from a CMV-immune subject. The concentration of each tetramer used in this study was individually determined, and was generally 40–50 µg/ml.

### Cytokine Flow Cytometry

PBMC were washed 2 times in serum free Ex-Vivo (BioWhittaker, Walkersville, MD) re-suspended at 10×10^6^ cells/ml in CFC media (Ex-Vivo +5 µg/ml CD28 +2.5 µg/ml CD49d (BD Biosciences, Immunocytometry Systems, San Jose, CA)). PBMC were plated at 1×10^6^ cells/well and incubated with PR1 (0.2 µM, 2 µM, 20 µM and 200 µM) or pp65-A2 (20 µM) in a total volume of 200 µL/well. Un-stimulated cells were used to set background and Staphylococcal Enterotoxin B (SEB, Sigma-Aldrich, St. Louis, MO), a non-specific T cell activator, was used at 5 µg/ml as a positive control. Plates were incubated at 37°C and 5% CO_2_ for 1 hour, Brefeldin A was added to a final concentration of 10 µg/ml and incubation continued for an additional 5 hours. Cells were then washed once in PBS and incubated in PBS +0.02% EDTA (Sigma-Aldrich, St. Louis, MO) at 37°C and 5% CO_2_ for 15 minutes. Cells were washed once with cold PBS and treated with 1x FACS Lysing Solution (BD Biosciences, Immunocytometry Systems, San Jose, CA) for 10 min at room temperature. Cells were centrifuged and treated with 1x FACS Permeabilizing Solution (BD Biosciences, Immunocytometry Systems, San Jose, CA) for 10 min at room temperature. Cells were then washed 2 times in wash buffer (PBS +1% BSA and 0.02% NaN_3_). Cells were incubated with anti-CD8, anti-IFNγ and anti-CD69 (both from BD Biosciences, Immunocytometry Systems, San Jose, CA) for 30 min at 4°C in the dark, washed 2 times in wash buffer and re-suspended in PBS +1% PFA. All samples were acquired within 24 hours of staining, using a FACScan flow cytometer and CellQuest software. At least 50,000 events were acquired and gating was done on small lymphocytes and CD8+ cells.

### T cell Phenotyping and Sorting

Titrated PR1/HLA-A2 tetramer was used to identify and distinguish between high and low avidity PR1-CTL, defined by a 1-log separation in fluorescence intensity [24]. 1×10^6^ PBMC were washed 3 times in PBS and surface stained with CD8 as well as CD14, CD16 and CD19 (all from Caltag Labs, Burlingame, CA) at 4°C for 30 minutes. CD14, CD16 and CD19 were used to exclude monocytes, NK cells and B cells from analyses. After washing, the cells were then incubated with 40–50 µg/ml of PE-labeled PR1/HLA-A2 tetramer at 4°C for 40 minutes, washed twice in PBS and fixed in 2% PFA. Cells were analyzed on a BD FACScan using CellQuest software. At least 80,000 events were collected.

For higher order multi-parameter phenotyping and cell sorting, the PBMC were thawed at 37°C, washed twice in PBS and re-suspended at 10×10^6^ cells/ml. A cocktail of appropriate antibody combinations of up to 8 unique colors from the following was used for staining cells: CD28, CD16 (Beckman Coulter, Miami, FL), CD8, CD14, CD16, CD19, CD45RA (Caltag Labs, Burlingame, CA), CD45RA, CD45RO, CD57, CCR7, CD27 (BD Pharmingen, San Diego, CA), CD4, CD45RA (BD Immunocytometry Systems, San Jose, CA) and CD45RA (Beckman Coulter, CA). When the multi-color combinations required it, CD45RA or CD27 was conjugated to Zenon™ Alexa350, or Zenon^™^ Pacific Blue (Molecular Probes, Eugene, OR). Cells were stained simultaneously with up to 8 antibodies in a cocktail for 25 minutes at 4°C in the dark. After washing twice in PBS, cells were stained with 40–50 µg/ml PE-conjugated PR1/HLA-A2-tetramer for 40 minutes at 4°C in the dark, washed twice in PBS and fixed in PBS +1% PFA. Single antibody stained cells were used to set compensation. Data was acquired on a MoFlo flow cytometer (Dako Cytomation, Fort Collins, CO), a Cyan flow cytometer (Dako Cytomation, Fort Collins, CO), or a FACSAria cytometer (BD Immunocytometry Systems, San Jose, CA) and analyzed using FlowJo analysis software (Treestar Inc, San Carlos, CA). Cell sorting was done on a FACSAria cytometer using the FACS Diva Software (BD Immunocytometry Systems, San Jose, CA).

### CFSE Cell Proliferation Assay

Cell proliferation was measured using carboxyfluorescein diacetate succinimidyl ester (CFSE, Molecular Probes, Eugene, OR) labeling. A 10 mg/ml stock solution of CFSE was prepared in dry DMSO (Sigma-Aldrich, St. Louis, MO) and stored at −20°C in 10 µL single-use aliquots. A working solution was prepared by diluting the stock solution with 990 µL dry DMSO. Up to 2.5×10^6^ PBMC or sorted cells were washed with PBS and re-suspended in 1 ml PBS at room temperature. 15 µL of 10 µg/ml CFSE was added and cells were incubated at 37°C for 8 minutes. Labeling was quenched with FBS. Cells were washed in PBS and incubated in media (Ex-Vivo+10%AB) for 5 minutes at 37°C. Cells were then washed with media, re-suspended in 1 ml media and stimulated with 20 µM PR1, or 50 ng/ml purified anti-CD3 (OKT3, Ortho-Biotech, Bridgewater, NJ) and 1 µg/ml anti-CD28 (BD Biosciences). IL-2 (R&D Systems) was added at 100 IU/ml to each well and un-stimulated cells were used to define CFSE intensity in undivided cells. Cells were incubated at 37°C and 5% CO_2_ for 80 hours and analyzed for proliferation by FACS. For analysis of surface phenotype after proliferation, cells were washed once with PBS and incubated with previously titrated amounts of antibodies to CD4, CD14, CD16, CD19, CD8, CD45RA, CCR7 and CD25 for 30 min at 4°C. After washing twice in PBS, cells were stained with 40–50 µg/ml PE-conjugated PR1/HLA-A2-tetramer for 40 minutes at 4°C in the dark, washed twice in PBS and fixed in PBS +1% PFA. Cells were acquired on a FACSAria cytometer or a Cyan cytometer. Single stained controls were used to set compensation and the data was analyzed using FlowJo. Cell divisions are measured by successive 2-fold dilution in CFSE intensity and represent a 2-fold increase in frequency. To estimate the frequency of cells that proliferated, the proportion of cells in each CFSE dilution peak was divided by 2^n^ (where n is the number of the cell division) and summed. To calculate proliferation index or average number of divisions per cell, the frequency of cells that proliferated in each division was normalized to 1, multiplied by the division number and summed.

### Statistical Analysis

A paired, two-tailed Student's t-test (95% confidence interval) was used to determine statistical significance of differences between samples. All data are represented as mean ± standard error.

## Results

### PR1-CTL persist in IFN-sensitive CML patients after IFN withdrawal

Previously, we have shown that PR1-CTL are found in most patients that achieve remission while being treated with IFN and that these PR1-CTL contribute to remission by killing CML cells [16]. Since 1–3% of patients that achieve remission do not relapse after IFN has been withdrawn, we hypothesized that PR1-CTL may contribute to the maintenance of remission in patients who remain in cytogenetic remission after IFN withdrawal. In order to determine whether PR1-CTL persist in the peripheral blood of patients that remain in remission after IFN withdrawal, we retrospectively studied available PBMC from nine HLA-A2+ CML patients that were treated with IFN 3-times weekly as first-line therapy. Patient characteristics at time of diagnosis are shown in **Table 1**. All patients were diagnosed with chronic phase CML, and four patients had intermediate or high Sokal scores.

Seven patients achieved CCR, then IFN was withdrawn and patients received no maintenance therapy. Three patients subsequently developed cytogenetic relapse (patients 2, 6, and 7) and were treated with imatinib, as summarized in **Table 2**. Two patients (patients 8 and 9) did not achieve CCR while receiving IFN. Complete molecular remission was noted in patients 3 and 4, and increased circulating PR1-CTL persisted during the study period (12–15 months).

**Table 2:** Patient disease responses and PR1-CTL immune responses.

Patient number	Time on IFN prior to withdrawal (mo)	Time since IFN withdrawal (mo)	BCR-ABL transcripts (IS, %)	PR1-CTL (% of CD8)	% of IFNγ+ CD8 cells (+PR1)	% of IFNγ+ CD8 cells (+SEB)
1	42	18	0.39	0.3	—	—
		26	0.077	0.74	—	—
2	80	15	0.035	0.53	0.18	4.97
		21	0.22	0.19	<0.01	7.96
		26*	8.1	<0.01	<0.01	6.34
		32*	0.67	0.25	0.1	3.89
		38*	0.066	2.17	—	—
3	98	45	0	0.83	2.63	7.04
		53	0	0.72	0.05	1.58
		60	0	2.11	0.06	3.45
4	108	75	0	0.89	0.06	5.21
		88	0	0.78	0.08	2.31
5	82	12	0.0039	2.73	2.2	15.27
		24	0.0041	1.53	2.5	21.59
6	69	0	0.088	0.33	—	—
		6	0.0011	1.46	3.83	14.09
		14*	2.4	0.03	<0.01	14.6
7	50	3	0.21	0.15	0.1	4.66
		11*	11.3	0.02	<0.01	5.27
		14*	0.16	0.31	0.19	6.2
8	8	NA	26.5	<0.01	—	—
	14	NA	21.4	<0.01	—	—
9	6	NA	11.6	0.02	—	—
	9	NA	15.7	<0.01	—	—

*Patient receiving imatinib 400 mg daily.

−Denotes that the result that could not be determined due to an insufficient number of cells.

NA Not applicable since IFN was not withdrawn in these patients.

BCR-ABL transcripts were determined with quantitative real-time PCR (RQ-PCR) of peripheral blood, as expressed on the International Scale (IS). PR1/HLA-A2 tetramers were used to enumerate PR1-CTL in PBMC, and PBMC were left unstimulated or stimulated with either the PR1 peptide or with SEB and analyzed for IFNγ production by flow cytometry. Shown are the frequencies of CD8+ T cells that produce IFNγ, after subtracting percentage of CD8+ cells that stain positive for IFNγ without any stimulation. The highest frequency of CD8+ cells that produce IFNγ when stimulated with PR1 are shown.

**Figure 1:**
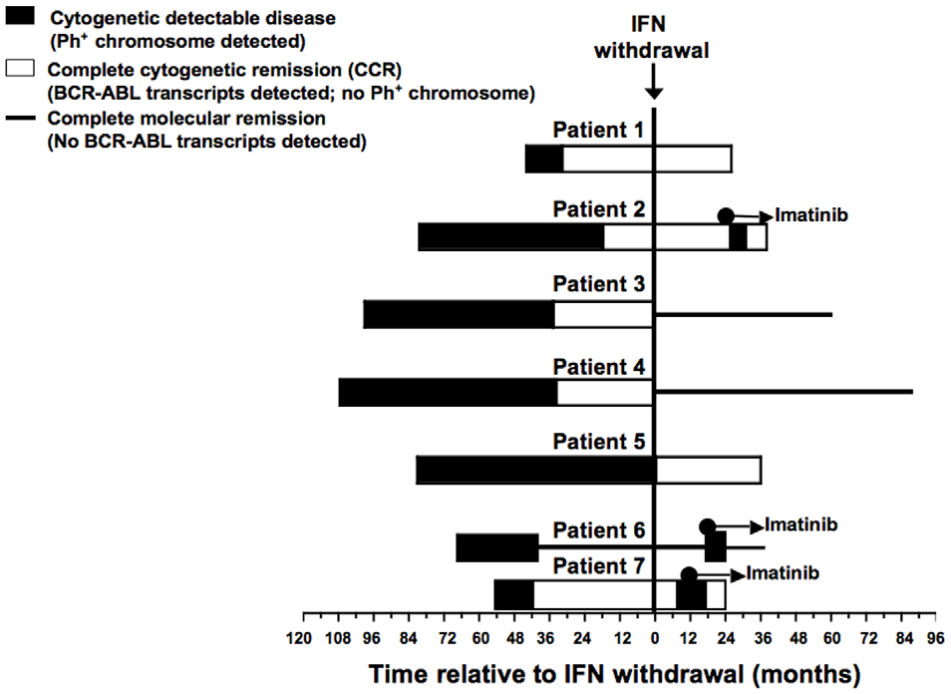
Longitudinal disease burden of CML patients in complete cytogenetic remission (CCR) after withdrawal of IFN treatment. Disease characteristics of seven HLA-A2+ patients in whom IFN treatment was withdrawn. Patients received IFN starting from the time of diagnosis (left-most time point) until it was stopped (denoted as time = 0). For each patient, closed bars denote time points when any Ph+ chromosome was detected in bone marrow, open bars denote time points when no Ph+ chromosomes were detectable (complete cytogenetic remission, CCR) but BCR-ABL transcripts were detectable by QR-PCR, and the solid line denotes time points when BCR-ABL transcripts were not detectable (complete molecular remission). Arrows denote when certain patients received imatinib monotherapy.

**Figure 2:**
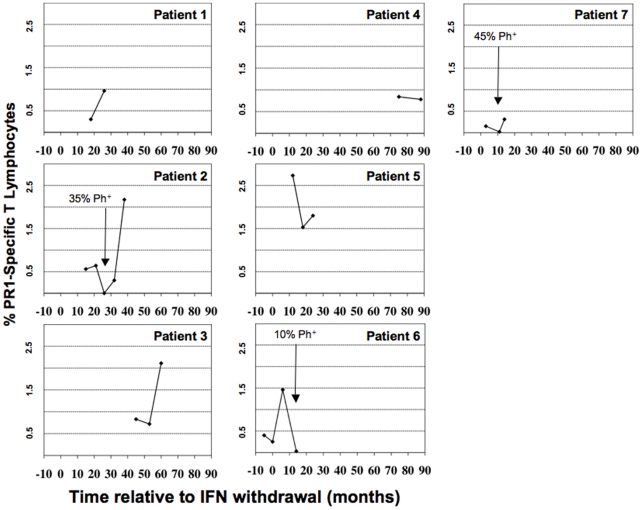
PR1-CTL persist in patients that maintain remission after IFN withdrawal. For each patient, the panel shows the total percentage of CD8+ cells that were identified with the PR1/HLA-A2 tetramer at time points after IFN withdrawal.

**Figure 1** shows the clinical status of the seven HLA-A2+ patients over time relative to the date of IFN withdrawal. From available archived samples, PBMC were stained with CD8, CD14, CD16, and CD19 antibodies, followed by staining with the PR1/HLA-A2 or pp65/HLA-A2 tetramers. All patients in cytogenetic remission (patients 1, 2, 5, and 7) and all patients in molecular remission (patients 3, 4 and 6) had detectable PR1-CTL in their peripheral blood (**Table 2**). The frequency of PR1-CTL as a percentage of CD8+ cells relative to time after IFN withdrawal is shown in **Figure 2** for each of the seven patients studied. At the time of cytogenetic relapse (patients 2, 6, and 7), the previously detectable PR1-CTL were no longer detectable.

### PR1-CTL are functional and secrete IFNγ

In order to assess whether the PR1-CTL were functional, when sufficient cells were available for analysis we incubated PBMC in media overnight prior to stimulation with PR1 peptide and measured IFNγ production by CD8+ T cells using cytokine flow cytometry in a 6 hour assay. Corresponding to time points after IFN withdrawal for each patient, **Table 2** shows the disease burden by *BCR-ABL* transcripts and the frequencies of PR1-CTL and of CD8+ cells that produce IFNγ when stimulated with either PR1 or SEB. Cells were stimulated with 4 different PR1 concentrations and the table shows the highest frequency of cells producing IFNγ. All patients that maintained remission (patients 3, 4, and 5) maintained a measurable functional response to PR1 at all times sampled after IFN withdrawal.

### PR1-CTL express all T cell differentiation phenotypes

The pattern of lineage differentiation phenotype of antigen-specific T cells has been shown to proceed from CD8^+^CD45RA^+^CCR7^+^ -> CD8^+^CD45RA^−^CCR7^+^ -> CD8^+^CD45RA^−^CCR7^−^ -> CD8^+^CD45RA^+^CCR7^−^, though this pattern may in part depend upon the antigen specificity of the CTL. This final stage of maturation has been associated with a non-proliferating terminally differentiated population [30]. However, antigen-specific CD8^+^CD45RA^−^CCR7^−^ T cells can re-express both CD45RA and CCR7, acquiring either the CD8^+^CD45RA^+^CCR7^+^ (naïve) or the CD8^+^CD45RA^−^CCR7^+^ (central memory) phenotype upon antigen stimulation, suggesting that there is a memory T cell population that can undergo expansion and ensure continuous replenishment of the effector cell pool. Furthermore, although the phenotype of the population of CD8+ T cells with replication senescence is not well described [31], [32], CD57 expression without CD28 or CCR7 expression has been shown to define terminally differentiated T cells [33].

**Figure 3:**
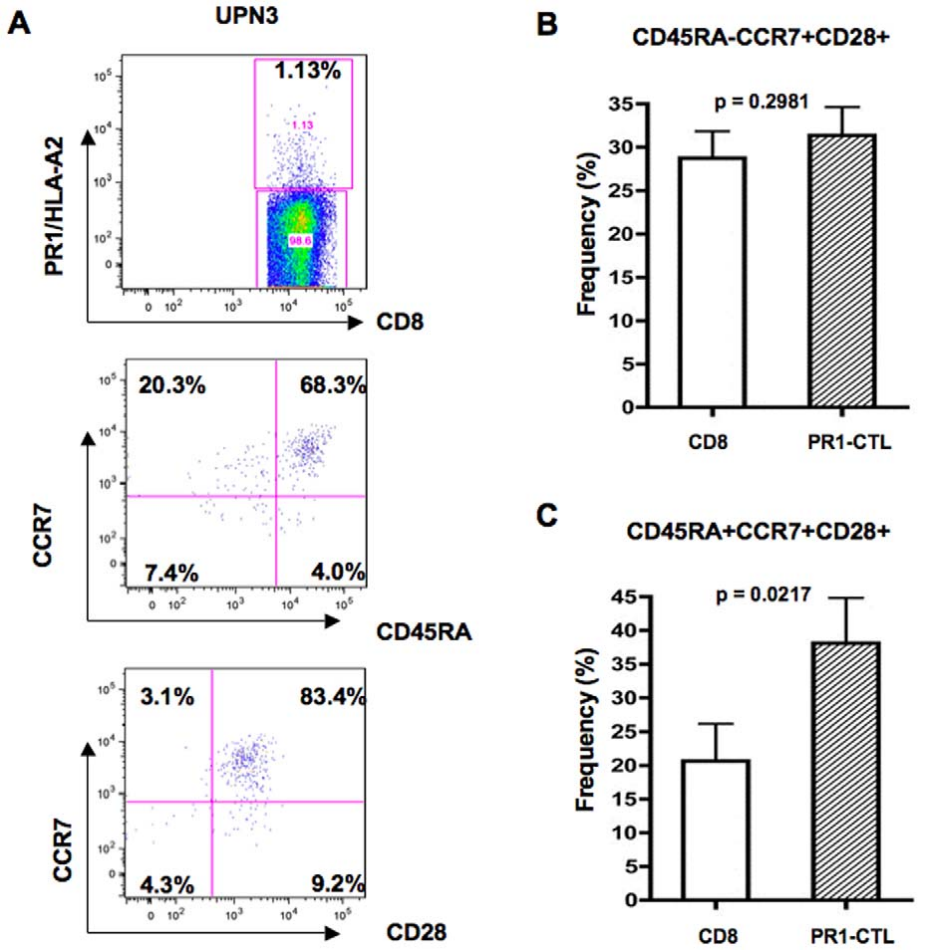
PR1-CTL express all T cell differentiation phenotypes. A cocktail of antibodies with 8 fluorochromes was used to label PBMC with anti-CD8, lineage markers (Lin; anti-CD4, anti-CD14, anti-CD16, anti-CD19), CCR7, CD27, CD45RA, CD28, CD57 and PR1/HLA-A2 tetramer. The data was analyzed using the FlowJo analysis software. (A) Gating was done on CD8+ Lin- lymphocytes in the upper panel and on PR1-CTL in the center and lower panels. The center and lower panels show the representative phenotype of naïve and memory PR1-CTL from patient 3. The percentage of PR1/HLA-A2 tetramer-negative and PR1/HLA-A2 tetramer-positive CD8+ cells that express (B) central memory or (C) naïve phenotype averaged from patients 1 to 7 is shown. P-values were calculated using a two-tailed t-test and results are shown as mean ± SEM.

To determine whether all lymphocyte differentiation phenotypes were present, we assessed the surface phenotype of PR1-CTL from all of the patients. **Figure 3A** shows a representative PR1-CTL phenotype from patient 3, 60 months after IFN treatment was stopped. Shown in **Figure 3B** and **3C** are the percentage of PR1/HLA-A2 tetramer-negative CD8^+^ lymphocytes (CD8+) and the PR1/HLA-A2 tetramer-positive CD8^+^ (PR1-CTL) that express central memory (CD45RA-CCR7+CD28+) or naïve (CD45RA+ CCR7+CD28+) phenotypes, averaged from all of the patients. Although there was no significant difference in the percentage of CD8+ lymphocytes and PR1-CTL that expressed the central memory phenotype (p = 0.2981), the PR1-CTL population was enriched for cells expressing the naïve phenotype as compared with the overall CD8+ lymphocyte population (p = 0.0217). Furthermore, none of the PR1-CTL expressed CD57 in any of the patients studied (data not shown). Since PR1-CTL did not express CD57 and were capable of producing IFNγ when stimulated with the PR1 peptide, together these data show that PR1-CTL from these patients are not anergic.

### PR1-CTL with central memory phenotype retain self-renewal capacity

**Figure 4:**
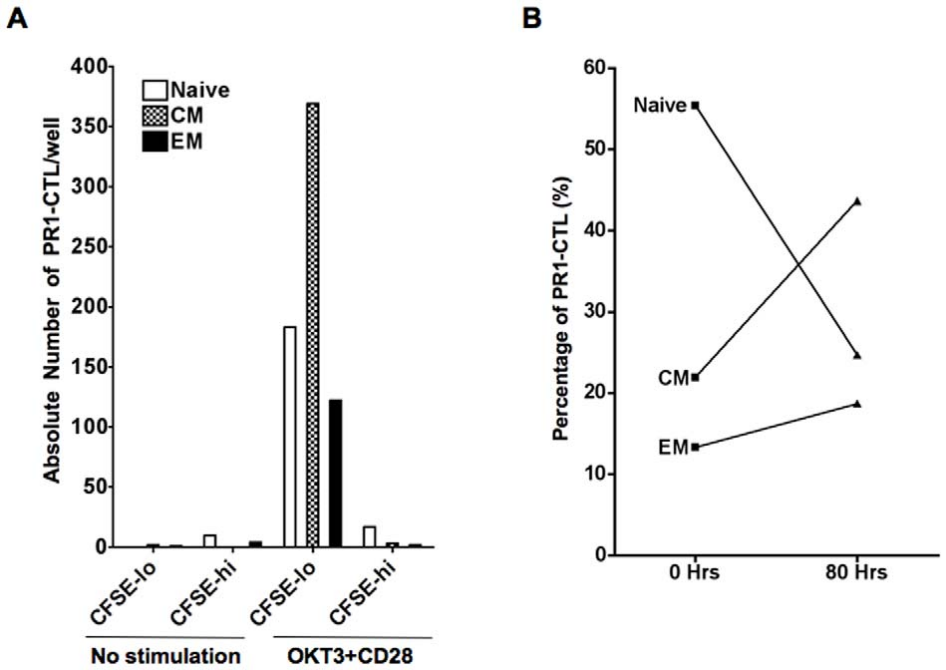
The phenotype of PR1-CTL changes from predominantly naïve to memory upon stimulation. PBMC from patient 3 were labeled with CFSE, stimulated for 80 hours, then labeled with antibodies for analysis of surface phenotype by FACS to determine naïve (N), central memory (CM), and effector memory (EM) cells, as described in the text. (A) The left panel shows the absolute number of PR1-CTL that proliferate after T cell receptor (TCR) stimulation with OKT3 and anti-CD28 antibodies. (B) The right panel depicts the percentage of PR1-CTL that were phenotypically categorized as N, CM, or EM before (time 0) and after (time 80 hrs) TCR stimulation. Prior to stimulation, PR1-CTL were predominantly naïve (N) phenotype and following stimulation PR1-CTL were predominantly CM phenotype.

A discrete population of PR1-CTL from all patients express a phenotype consistent with memory T cells. We hypothesized that PR1-CTL from IFN-sensitive CML patients consists of a self-renewing memory T cell population. To determine whether PR1-CTL could proliferate, we labeled PBMC from patient 3 with CFSE, added an equal number of cells per well and stimulated them with OKT3, anti-CD28 and IL-2. To assess the surface phenotype of proliferating PR1-CTL, the cells were labeled with the PR1/HLA-A2 tetramer and antibodies to CD8, CD45RA, CCR7 and CD25 after stimulation and analyzed by flow cytometry. **Figure 4** shows that in the absence of stimulation, PR1-CTL underwent cell death and could not be identified. However, with CD3/CD28/IL-2 stimulation, PR1-CTL proliferate robustly and the largest fraction of proliferating PR1-CTL express a phenotype consistent with central memory T cells. We compared the phenotype of PR1-CTL before and after stimulation and determined that although prior to stimulation a majority of PR1-CTL expressed a naïve T cell phenotype, after proliferation the PR1-CTL were predominantly central memory T cells (**Figure 4**).

**Figure 5:**
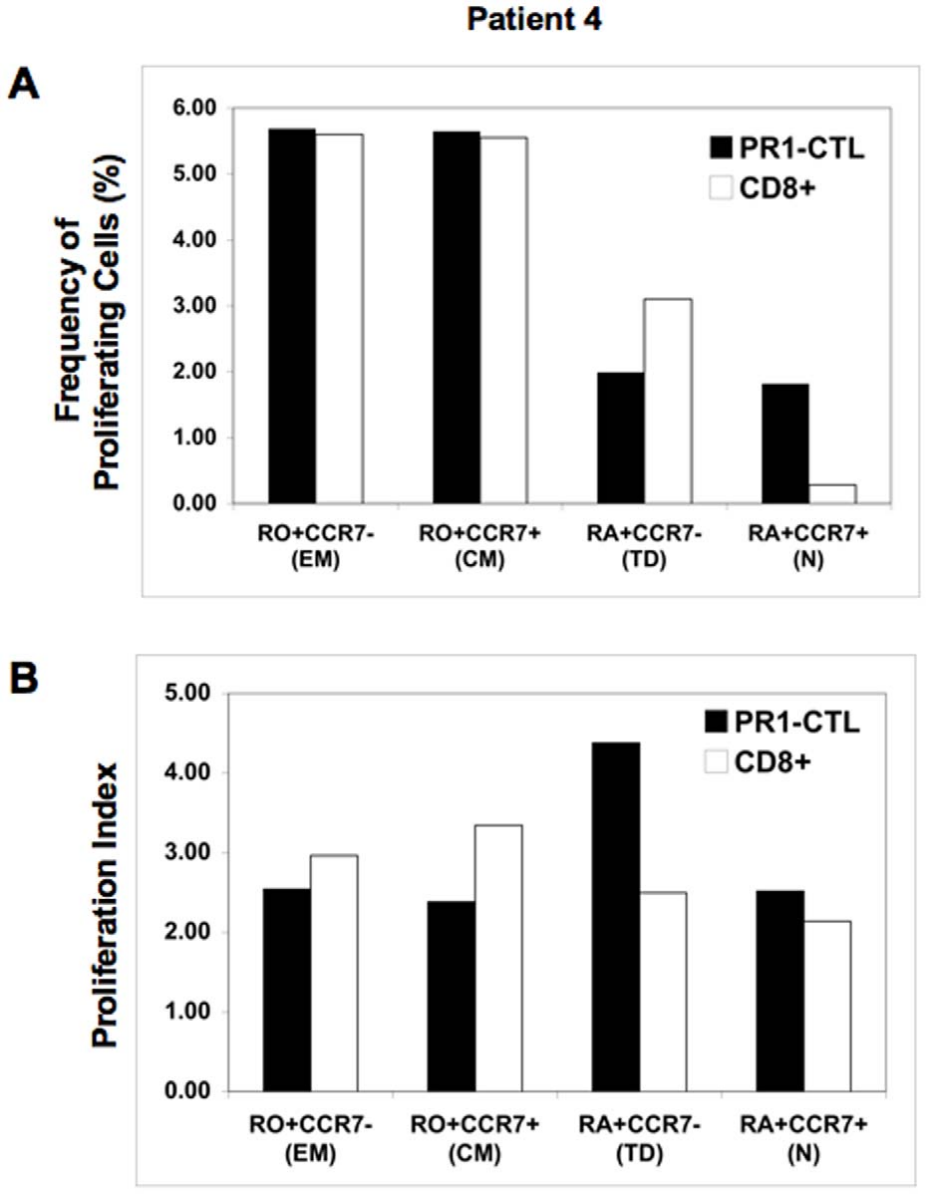
PR1-CTL of all differentiation phenotypes proliferate. Lin- CD8+ cells from patient 4 were sorted into effector memory (EM), central memory (CM), terminally differentiated (TD) and naïve (N) populations, then labeled with CFSE and stimulated with OKT3 + anti-CD28 antibodies. After 80 hours, cells were labeled with PR1/HLA-A2 tetramer and analyzed for evidence of proliferation by CFSE dilution. (A) The upper panel shows the frequency of proliferating PR1-CTL compared to total CD8+ T cells, and (B) the lower panel shows proliferation indices of PR1-CTL compared to total CD8+ T cells.

While the results in **Figure 4** support our hypothesis that memory PR1-CTL proliferate, labeling to identify phenotypes after proliferation may have biased the results toward cells that undergo multiple cell divisions or that re-express CCR7. Therefore, to determine whether the central memory PR1-CTL could proliferate, we sorted CD8+ T cells from patient 4 into 4 distinct differentiation phenotypes and measured proliferation of each. CD8+ T cells were sorted into the following populations: CD45RO+CD45RA-CCR7+ (central memory), CD45RO+CD45RA-CCR7- (effector), CD45RO-CD45RA+CCR7+ (naïve) and CD45RO-CD45RA+CCR7- (terminally differentiated). After sorting, cells were labeled with CFSE and stimulated for 80 hours. Cells were then stained with tetramer and analyzed for proliferation. For each cell population, the fraction of cells that diluted CFSE was analyzed and the frequency of cells that proliferated was calculated. We found that that the frequency of effector and central memory PR1-CTL that proliferated was similar to the frequency of the overall CD8+ that proliferated (**Figure 5A**). We also found that a higher frequency of naïve PR1-CTL proliferated as compared with the overall CD8+ population. We next compared the proliferation index or average number of divisions per cell of PR1-CTL with the overall CD8+ cells in each sub-population. **Figure 5B** also shows that the proliferation indices for the naïve, effector and central memory PR1-CTL were approximately 2.5 whereas the proliferation index for the terminally differentiated PR1-CTL was 4.4. Senescent T cells have been shown to express CD57 [33] and PR1-CTL from IFN-sensitive CML patients off all therapy do not express CD57 (data not shown), indicating that PR1-CTL with a terminally differentiated phenotype are not senescent. These data also suggest that there is a self-renewing PR1-CTL population that may contribute to maintaining remission in this subset of patients.

### Loss of functional PR1-CTL immunity occurs prior to cytogenetic relapse

**Figure 6:**
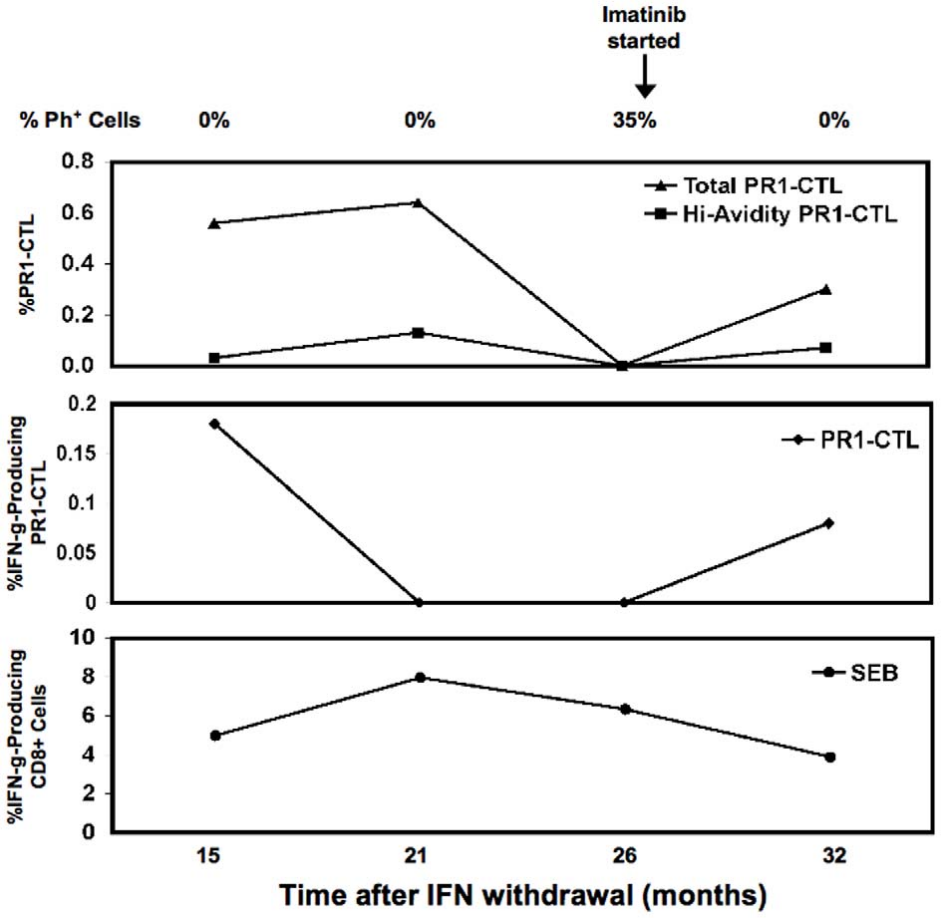
Loss of functional PR1-CTL precedes cytogenetic relapse in patient 2. Staining with the PR1/HLA-A2 tetramer was done to determine the percentage of high- and low-avidity PR1-CTL as described in the methods. In the top panel, PR1-CTL are defined as CD8+ Lin- PR1/HLA-A2-tetramer+. The center panel shows the percentage of CD8+ lymphocytes that produce IFNγ in response to stimulation with 0.2 µM PR1 peptide after subtracting the percentage of CD8+ cells that produced IFNγ in the absence of stimulation. Non-stimulated cells were used to adjust background. Stimulation with SEB superantigen was used to confirm that the lymphocytes were not anergic. The lower panel shows the frequency of CD8+ cells that produce IFNγ in response to SEB stimulation.

Patient 2 relapsed 26 months after IFN withdrawal. Following relapse, the patient was treated with imatinib (**Table 2**). We hypothesized that functional PR1-CTL would be observed prior to relapse that would be selectively lost at relapse. We assessed the persistence of PR1-CTL after IFN withdrawal by staining with PR1/HLA-A2 tetramer and determined whether the PR1-CTL were functional by assessing intra-cellular IFNγ production in response to stimulation with the PR1 peptide at varying concentrations. At 15 months after IFN withdrawal, patient 2 remained in CCR and had functional high and low avidity PR1-CTL (**Figure 6**) that exhibited a memory phenotype (as shown in **Figure 3**). Of the CD8^+^ cells, 0.18% showed IFNγ production in response to stimulation with 0.2 µg/ml PR1 peptide. At 21 months after IFN withdrawal, the patient remained in CCR and had 0.19% PR1-CTL. However, PR1-CTL did not produce IFNγ in response to stimulation with the PR1 peptide at concentrations varying from 0.2 µg/ml to 200 µg/ml of PR1 (**Figure 6**), although CD8+ T cell responses to SEB were maintained. This shows a selective loss of functional PR1-CTL. Five months later, at 26 months after IFN withdrawal, patient 2 had a cytogenetic relapse with 35% Ph^+^ cells and PR1-CTL were no longer detectable. Furthermore, PR1-CTL that were increased after stopping IFN were either not detectable or at the lower limit of detection in patients 6 and 7 at time of cytogenetic relapse (**Table 2**). Similarly, PR1-CTL were also not functional at the time of cytogenetic relapse (**Table 2**). Interestingly, after the three patients were started on imatinib, PR1-CTL once again increased and secreted IFNγ. In contrast, PR1-CTL were not detectable in patients 8 and 9 that did not achieve CCR during IFN therapy. Thus, patients who relapse after IFN withdrawal may preferentially loose functional PR1-CTL immunity prior to the decrease in total PR1-CTL.

## Discussion

Although direct effects of IFN on CML cells have been described as a mechanism of inducing remission, how this remission is maintained in the absence of continuous IFN treatment in patients with persistent molecular disease remains unknown. We hypothesized that IFN induces a self-renewing population of leukemia-specific CTL that contribute to remission in the absence of continuous therapy. We have previously shown that although the frequency of PR1-CTL in the peripheral blood of healthy HLA-A2+ donors are below the limits of detection by tetramer analysis, they can be expanded *in vitro* [34]. We have also shown that PR1-CTL are not detectable in newly diagnosed CML patients or in patients that do not respond to treatment with IFN and that PR1-CTL contribute to T cell mediated anti-CML immunity [16]. Furthermore, a recent study by Buchert et al demonstrated that PR1-CTL persist after withdrawal of imatinib in patients that continued to receive maintenance IFN and was associated with long-term CR [29]. In that study, PR1-CTL immunity was not assessed in two patients that achieved complete molecular remission in IFN maintenance. Here, we extend these observations and present three findings that provide evidence that functional memory PR1-CTL contribute to anti-leukemia immunity after IFN withdrawal. First, we show that PR1-CTL are maintained in IFN-sensitive CML patients for 6 months to 7 years after stopping all treatment. Second, we show that while all lymphocyte differentiation phenotypes are present in PR1-CTL, the CD45RA-CD28+CCR7±CD57- PR1-CTL expand preferentially after antigen stimulation, which is consistent with a self-renewing central memory T cell population. Finally, we show that PR1-CTL produce IFNγ in response to PR1 peptide stimulation in a recall response. In 3 patients that relapsed, however, PR1-CTL were unable to produce IFNγ prior to or concomitant with relapse of CML. Combined with previous studies showing PR1-CTL kill CML and contribute to CR in IFN-sensitive CML patients [16], our current study suggests that PR1-CTL expand during IFN therapy and that IFN may facilitate long-term persistence of PR1-CTL in a subset of CML patients that remain disease-free after all treatment has stopped.

Our data showing the persistence of functional PR1-CTL in patients 1 to 7 after IFN withdrawal extend the results of a previous study that showed measurable PR1-CTL by tetramer staining in 4/4 patients 3 years after cessation of IFN treatment [35]. Memory T cells are characterized by expression of the CD45RO isoform [36], but a fraction of memory cells express CD45RA [37], [38]. Previous studies with antigen-specific T cells for the CMV-derived pp65-A2 peptide have shown that upon stimulation, cell division is predominantly restricted to the CD8+CCR7+ population and that CD8+CCR7+ cells are also capable of secreting IFNγ upon antigen-specific stimulation, consistent with our findings. Furthermore, CD45RA-CCR7-CD8+ T cells that are at a more advanced stage of differentiation, though not senescent, can re-acquire CCR7 and/or CD45RA upon antigen-specific stimulation [30]. In our study, all patients that remained in remission had PR1-CTL that were enriched for CD8+CD45RA+CCR7+, with a smaller fraction that were CD8+CD45RA-CCR7+. In order to determine whether PR1-CTL were capable of dividing, we sorted the cells to >95% purity and stimulated them in a proliferation assay. Consistent with the characteristics of memory T cells, we found that the effector and central memory PR1-CTL proliferated robustly, supporting our hypothesis that IFN treatment induces a self-renewing population of leukemia-specific CTL that may help to maintain anti-leukemia immunity and remission in the absence of ongoing treatment. Interestingly, we found that a higher frequency of PR1-CTL with a naïve T cell phenotype proliferated as compared with the overall CD8+ cells while the average number of divisions undergone was similar to that of memory PR1-CTL (**Figure 5**). Although CD45RA+CCR7+ CTL includes naïve T cells, the high frequency of PR1-CTL in the peripheral blood of IFN-sensitive patients, the production of IFNγ in a recall response and the ability to proliferate strongly suggests that this may be a re-expanded population rather than an antigen-inexperienced one. Future studies should discern whether these phenotypically naïve cells are recent thymic emigrants or whether they come from self-renewing central memory stem cells [39] that re-express CD45RA. Since PR1 is a self-antigen derived from P3 and NE, proteins that are expressed in myeloid cells, the antigen required for maintaining this population may come from normal myeloid cells. Alternatively, PR1 might derive from residual small fraction of leukemia cells that over-express P3 or NE, or it is possible that the level of PR1 expression on non-leukemia cells is sufficient to maintain a PR1-CTL population.

The loss of function of PR1-CTL prior to relapse (**Figure 6**) suggests that broader anti-CML immunity might be lost prior to disease relapse. We were unable to address whether loss of function is a broad-based phenomenon that applies to other LAAs or whether this is restricted to the PR1 peptide antigen due to limited availability of patient material. However, we observed that at each time-point studied, CD8+ T cells produced IFNγ in response to stimulation with SEB, suggesting that loss of function is antigen-specific and might therefore apply to immunity against other LAAs. Following relapse, patients 2, 6, and 7 were treated with imatinib, achieved CCR, and PR1-CTL again increased. There are contradictory reports on the effect of imatinib on T cell function and although many investigators have described the immunosuppressive effects of imatinib on both T cells and dendritic cells [40], [41], [42], other reports indicate that imatinib does not induce apoptosis of T cells and that its inhibitory effects on T cell proliferation and function are reversible after removal of the drug [43], [44]. While enhanced IFNγ production by T cells has also been reported in IFN-refractory CML patients treated with imatinib [45], a more recent study showed distinct immunological status of patients treated with imatinib and IFN [46]. In our study, we observed that following treatment with imatinib, both PR1-CTL and pp65-specific CTL from patients 2, 6, and 7 produced IFNγ when stimulated. Since the cells were studied *in vitro*, we cannot rule out inhibitory effects of imatinib in vivo. However, our data are consistent with studies showing that imatinib does not induce T cell apoptosis [43] and that treatment with IFN after cessation of imatinib is associated with an induction of PR1-CTL [29].

Although the data from this study shows that functional immunity to PR1 persists during unmaintained remission of CML, it does not demonstrate that PR1-CTL immunity is the sole driver of sustained molecular remission. Because of limited availability of patient material, we did not study immunity to other known CML antigens such as WT1 or the BCR-ABL fusion region peptides, but we would expect that CTL immunity against these and perhaps other leukemia antigens should be similarly increased. Furthermore, because we did not demonstrate that PR1-CTL were directly cytotoxic against CML cells or against LSC in particular, it is possible that the observed changes in the number of PR1-CTL may merely reflect changes in the leukemia burden. For instance, we have shown that CML shapes host immunity by preferential elimination of high avidity PR1-CTL, which are more cytolytic against CML than low avidity PR1-CTL, and the data in **Figure 6** support these previous observations [24].

In summary, our data provide evidence that PR1 leukemia antigen-specific CTL-mediated anti-tumor immunity after IFN withdrawal may contribute to continued CR in the absence of subsequent treatment, including withdrawal of IFN. This study extends the observations of Buchert et al and suggests that long-term persistence of tumor-reactive CTL may be necessary to control the outgrowth of residual leukemia cells and prevent relapse. Subsequent prospective studies should examine whether loss of functional PR1-CTL immunity predicts relapse. Strategies to boost immunity by vaccination with known leukemia-antigens in combination with IFN or strategies for IFN maintenance therapy after imatinib treatment, as suggested by the results of a prospective clinical trial in patients treated with imatinib and IFN [29], might improve responses in patients.
